# Primary Intrarenal Neuroblastoma with Hypertension and Disseminated Intravascular Coagulation

**DOI:** 10.1155/2013/684939

**Published:** 2013-12-12

**Authors:** Bibi Shahin Shamsian, Mohammad Kajizadi, Nima Rezaei, Nozar Ghojehvand, Roxana Azma, Mohsen Rouzrokh, Maryam Kazemi Aghdam, Seyed Malek Mesbah, Farid Ghazizadeh, Mohammad Taghi Arzanian

**Affiliations:** ^1^Pediatric Congenital Hematologic Disorders Research Center, Mofid Children's Hospital, Shahid Beheshti University of Medical Sciences, Tehran 15468-15514, Iran; ^2^Department of Pediatric Hematology-Oncology, Mofid Children Hospital, Shariati Avenue, Tehran 15468-15514, Iran; ^3^Research Center for Immunodeficiencies, Children's Medical Center, Tehran University of Medical Sciences, Tehran 14194, Iran; ^4^Molecular Immunology Research Center, School of Medicine, Tehran University of Medical Sciences, Tehran 14194, Iran; ^5^Department of Immunology, School of Medicine, Tehran University of Medical Sciences, Tehran 14194, Iran; ^6^Department of Pediatric Radiology, Mofid Children's Hospital, Shahid Beheshti University of Medical Sciences, Tehran 15468-15514, Iran; ^7^Department of Pediatric Surgery, Mofid Children's Hospital, Shahid Beheshti University of Medical Sciences, Tehran 15468-15514, Iran; ^8^Department of Pediatric Pathology, Mofid Children's Hospital, Shahid Beheshti University of Medical Sciences, Tehran 15468-15514, Iran

## Abstract

The primary intrarenal neuroblastoma (IRNB) is a rare condition. Intrarenal neuroblastoma typically results from direct renal invasion from an adrenal neuroblastoma, but true intrarenal neuroblastoma originates either sequestered adrenal rests during the fetal life or intrarenal sympathetic ganglia. Clinical, radiological, and pathological correlation is very essential for diagnosis and appropriate management of this type of unusual cases. The distinction of this rare tumor from Wilms' tumor is an important challenge since both tumors have major differences in prognostic and therapeutic response. We present a 3-year-old boy of primary intrarenal neuroblastoma with extensive abdominal and mediastinal mass, persistent hypertension, and disseminated intravascular coagulation (DIC).

## 1. Case Report

**Figure 1:**
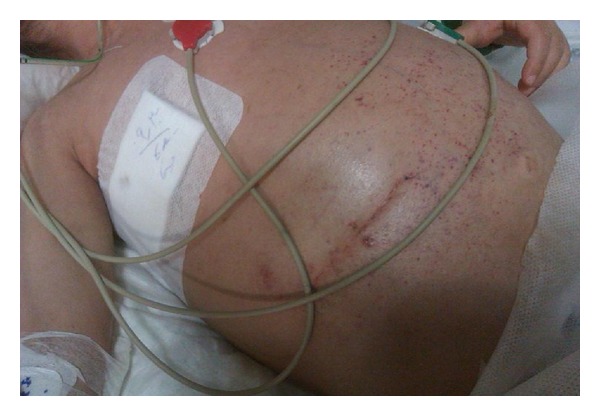
Large abdominal mass and petechiae, purpura due to DIC in patient with neuroblastoma.

A 3-year-old boy was referred to our hospital with nausea and vomiting since 2 months before admission and massive abdominal mass in the right upper quadrant of abdomen, extending up to the midline. Physical examination revealed a unilateral, palpable, firm, abdominal mass extending to more than 10 cm below the right costal margin (Figure 1).

**Figure 2:**
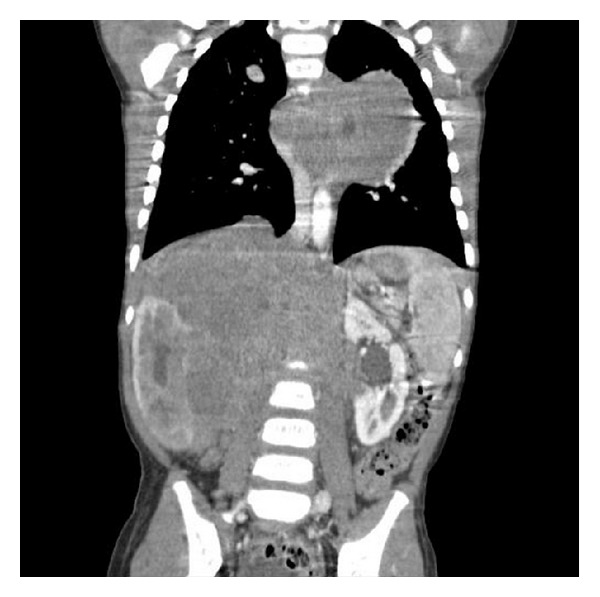
CT scan of abdomen and chest; large abdominal mass in right side and paravertebral mediastinal mass in left side.

**Figure 3:**
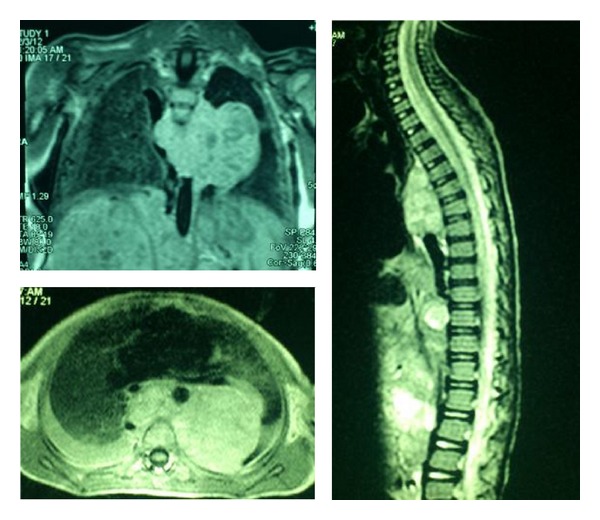
MRI showing left paravertebral mediastinal mass without intracanal invasion.

The results of initial laboratory tests were as follows: WBC = 5100, PMN = 52%, lymph = 45%, Hb = 8, platelets = 109000, LDH = 15714, ESR = 7, uric acid = 6, BUN = 15, creatinin: 0.7, SGOT = 260, SGPT = 25, bilirubin total = 2.2, bilirubin direct = 0.9, PT = 13, and PTT = 36. Bone marrow aspiration and biopsy were normal. Bone survey showed permeative lesions, some erosion in distal of right femur and lateral of middle right tibia, but bone scan was normal. Abdominal ultrasound revealed a large abdominal mass. So, computed tomography (CT) scan of abdomen and pelvis for more evaluation was performed, which showed an extensive tumoral lesion, size of 110 × 56 × 90 mm (intracapsular) in right kidney with dilatation of calyceal system, some deformity, and some scattered calcification. The tumor extended beyond the abdomen midline till the left paravertebral region, while bilateral adrenals were not seen. Hydronephrosis and some deformities of right kidney were seen. Left kidney was normal (Figure 2). Magnetic resonance imaging (MRI) of thorax showed bilateral pleural effusion. Large lobulated paraspinal soft tissue lesion was seen, which was extended from the abdomen upward into the thoracic cavity. In thorax, a significant bulk was seen in the left side of thoracic spine, which had encased thoracic aorta and had displaced azygos vein, involving proximal part of some left ribs. Large lobulated soft tissue mass lesion of abdominal cavity with enhancement of abdominal vessels was also seen, but no intracanal extension was noted (Figure 3). Brain CT scan was normal.

**Figure 4:**
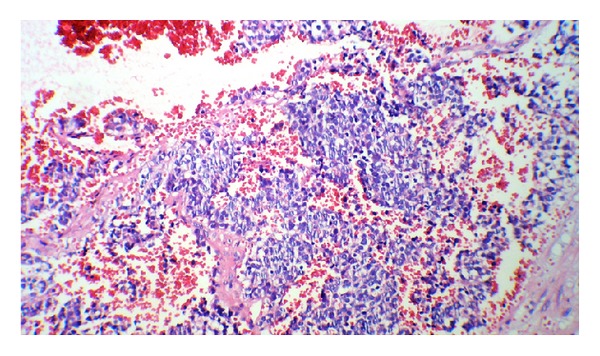
Small round neoplastic cells with hyperchromatic nuclei.

**Figure 5:**
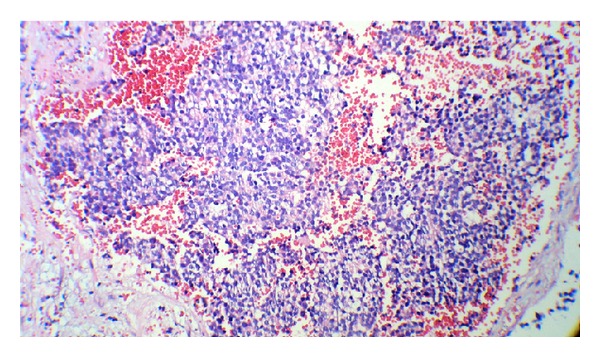
Small round cell tumor in hemorrhagic fibrovascular background.

**Figure 6:**
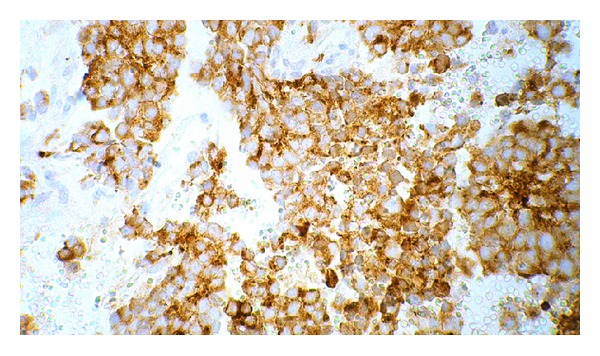
Chromogranin immunohistochemistry in tumor cells.

**Figure 7:**
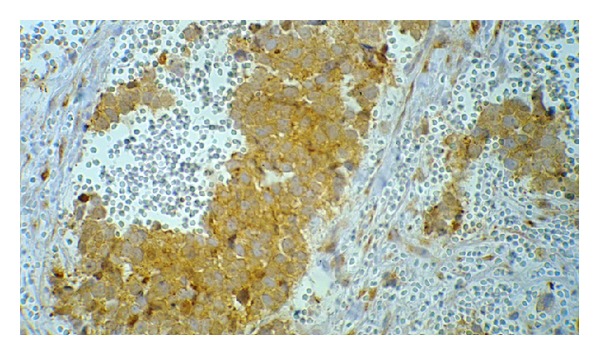
Synaptophysin immunohistochemistry in tumor cells.

On day 2 of admission, laparatomy was done; tumor was associated with massive hemorrhage, and just biopsy was done. After surgery, he suffered from persistent hypertension. So, treatment was started to control the blood pressure. The results of histopathology report indicated neuroblastoma with poor stroma, poor differentiation, and MKI < 100/5000 neuroblast: unfavorable (Figures 4 and 5). Immunohistochemistry (IHC) revealed NSE, Chromogranin, Synaptophysin, positive (Figures 6 and 7). Based on histopathology and staging of tumor diagnosis of neuroblastoma, stage 4 was made. Evaluation for N-Myc gene amplification revealed 420 copy number. Chemotherapy was started on day 6 after surgery with protocol N6 (Course 1: VCR, Cyclophosphamide, Adriamycin).

On day 6 of after surgery, the patient developed dyspnea, pleural effusion; therefore, pleural tap was done and then chest tube was inserted. He suffered from massive bleeding. Evaluation for bleeding disorders was in favor of overt DIC: fibrinogen = 140, FDP > 320, D-Dimer = 32/9, low platelet count, and long PT and PTT. He received supportive therapy, including packed red blood cell, platelet, FFP, cryoprecipitate, and Novoseven because of continous bleeding. Finally, bleeding was stopped. Now, patient is clinically stable. Our plan for this child is chemotherapy with N6 protocol, second-look surgery, and then autologous stem cell transplantation based on stage of disease, unfavorable histopathology report, and N-Myc gene amplification result.

## 2. Discussion

Primary IRNB is a rare clinical entity [1]. Lall et al. reported in 2001 that renal invasion by neuroblastoma occurs by direct penetration through the renal capsule and/or lymphatic perivascular spread [2]. Renal invasion occurs in approximately 20.4% of cases of abdominal neuroblastoma. A high proportion of intrarenal neuroblastoma are of unfavourable histology as defined by the International Neuroblastoma Pathology Classification and have a higher incidence of anaplasia (32%) when compared to both their adrenal counterparts and to Wilms' tumor [3].

A higher incidence of hypertension (66–100%) has been associated with intrarenal neuroblastoma as compared to 27% reported in the literature for neuroblastoma, probably because of compression of renal vessels, increased renin release from the kidney, and a high circulating level of catecholamines. Lall et al. reported hypertension in all their cases of intrarenal neuroblastoma [2]. Urinary catecholamines could be high in this group of patients or may be negative [2]. Most of these patients are metastatic at presentation (bone, bone marrow). Our patient showed involvement in bone skeletal, but bone scan was normal. So we decided stage 4 for him.

About primary intrarenal neuroblastoma, Fan (2012) wrote, if the tumor primarily involves the kidney without evidence of involvement of other primary sites, then it is considered a primary renal tumor. It is possible, and in fact speculated, that some so-called primary renal neuroblastomas actually arose from the immediate pararenal/hilar region, which, for all practical purposes, should be managed in the same or similar manner [4]. The factors that affect survival are age and health of child, extent of the disease, size, type and location of the tumor, metastasis, tumors response to therapy, and overall child's tolerance to medications [3].

There are some reports about association of neuroblastoma and disseminated intravascular coagulation (DIC) [5–7]. Our patient based on imaging shows a case of primary intrarenal neuroblastoma with persistcnt hypertension and unfavorable histopathology. Our patient had a vascular tumor with massive hemorrhage during laparatomy and also had evidnces of overt DIC during of his treatment. In article by Fan: A quick search of reports found a total of 380 patients registered in their institution during the same 15-year period (1993 through 2011) who were diagnosed with neuroblastoma. Of these neuroblastoma patients, 231 were from the abdominal area. For some cases, even though the kidney specimens clearly simulated Wilms' tumor on gross examination, they were excluded from the study secondary to the knowledge of other abdominal or retroperitoneal extrarenal sites involvement on the basis of the surgeon's input or radiologic data—resulting in the final count of 8 cases. This puts the incidence of primary renal neuroblastoma cases in the proximity of 1% to 2%, which is comparable with 11 neuroblastoma cases simulating Wilms' tumor reported in Shamberger et al.'s paper, of 868 cases in a 10-year span. So the frequency is higher than originally expected; nonetheless, the 8-case collection from a single institution does point to the belief that the incidence of renal neuroblastoma is perhaps higher than it was generally believed.

## 3. Conclusion

Primary intra renal is an extremely infrequent tumor in kidney. Clinical, radiological, and pathological correlation is very essential for diagnosis and appropriate management of this type of unusual cases. The distinction of this rare tumor from Wilms' tumor presents an important challenge since both tumors have major differences in prognostic and therapeutic response.
